# Re-emergence of vaccine-preventable diseases in the post-elimination era

**DOI:** 10.1371/journal.pmed.1005175

**Published:** 2026-07-16

**Authors:** Chirag K. Kumar, Nathan C. Lo

**Affiliations:** 1 Division of Infectious Diseases and Geographic Medicine, Department of Medicine, Stanford University, Stanford, California, United States of America; 2 Emmett Interdisciplinary Program in Environment and Resources, Doerr School of Sustainability, Stanford University, Stanford, California, United States of America

## Abstract

Measles and other vaccine-preventable diseases are re-emerging worldwide in locations where they were once eliminated. In this Perspective, Chirag Kumar and Nathan Lo outline how and why targeted policy interventions in the locations at greatest risk may be an effective strategy to curb this public health threat.

## Teaser

Measles and other vaccine-preventable diseases are re-emerging worldwide in locations where they were once eliminated. Targeted policy interventions in the locations at greatest risk may be an effective strategy to curb this public health threat.

In recent years, vaccine-preventable diseases have resurged in locations where they had been previously eliminated, in some cases leading to re-established local endemic transmission. This challenge of re-emergence is being experienced across numerous pathogens and around the world. Most notably, measles has resurged globally [1] and poses the greatest threat of widespread re-emergence among vaccine-eliminated diseases. In 2025−2026 alone, seven countries across three continents lost their World Health Organization measles elimination status [2]. The measles-mumps-rubella (MMR) vaccine has been the target of considerable vaccine misinformation and politicised discussions, with consequential vaccine hesitancy and reduced vaccine uptake [3,4]. More generally, there have been broad global declines in vaccine coverage, due to disruptions in immunisation programs from the COVID-19 pandemic, access barriers, and hesitancy. Collectively, these factors have increased the risk for outbreaks and re-emergence of vaccine-eliminated diseases, which will result in preventable illness and death [5], disproportionately among children.

Beyond measles, other vaccine-preventable diseases are also at risk of re-emergence. Although subsequently contained, an outbreak of wild poliovirus type 1 occurred in Southeast Africa in 2021–2022 after decades without outbreaks in this region and the African continent being declared free of wild poliovirus in 2020 [6]. Across West Africa, a significant multi-country diphtheria outbreak is occurring amidst declining immunisation levels, negating progress made towards elimination across the region [7]. Diphtheria outbreaks are often associated with conflict settings leading to migration, healthcare disruptions, and immunisation gaps [8]. Curbing the re-emergence of vaccine-preventable diseases will require targeted, context-specific interventions in the locations at greatest risk of outbreaks and collaborative partnerships between public health practitioners, clinicians, scientists, community leaders, and policymakers to address this growing threat.

Re-emergence risk varies across vaccine-preventable diseases [9]. For diseases that are more transmissible (measured by the basic reproduction number, R_0_, which is the average number of secondary cases generated by a single infected person in a fully susceptible population), a greater degree of population immunity is required to ensure that infection re-introduction does not lead to sustained transmission. Measles (with R_0_ of 10–20) requires very high population immunity to prevent sustained transmission compared to, for example, rubella, which has an R_0_ of 4–7 and can remain eliminated at a comparatively lower level of population immunity [9]. Additionally, re-emergence requires re-introduction of the infection, often related to travel from an endemic country. Since the incidence of each infectious disease differs substantially around the world, each infection has a different likelihood of re-introduction; for example, measles is common while poliovirus is rare [9]. Together, this underscores that re-emergence risk depends on multiple factors, including population immunity (which is shaped by historical vaccine uptake and effectiveness), disease transmissibility, and likelihood of re-introduction (which is related to global incidence).

**Fig 1:**
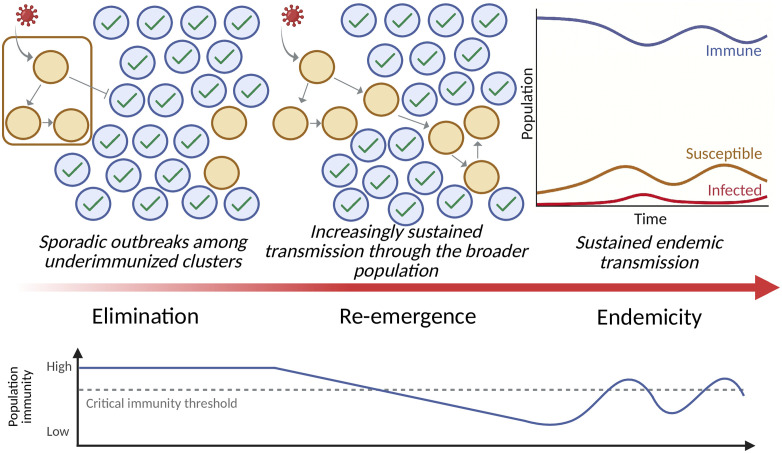
Schematic of transition from elimination to re-emergence to endemic transmission of a vaccine-preventable disease. Re-emergence occurs when a disease is introduced into a population that has become sufficiently susceptible to sustain transmission, which in some cases can lead to re-established endemic transmission. In the re-emergence stage, outbreak risk is highly variable and driven not only by population immunity but also by population structure, mobility, and public health response. Whether an imported index case triggers a sustained outbreak is stochastic, depending on with whom and how they interact with social contacts, when their infection is detected, and the public health response. As transmission becomes sustained, there is a transition to endemicity. Orange circles represent under-immunised individuals while blue circles represent immunised individuals.

The re-emergence of a vaccine-eliminated disease follows different epidemiologic patterns than the emergence of a novel infectious disease, leading to spatially concentrated outbreak risk (Fig 1). While eliminated, sporadic outbreaks of a vaccine-preventable disease may still occur among clusters of under-immunised individuals, most often triggered by an imported travel-associated infection. As vaccination rates decline, such outbreaks become increasingly sustained by infections that also occur among the broader local population. Re-emergent outbreaks occur when there is both high importation and high susceptibility, meaning that risk is greatest in a subset of locations. For instance, despite US measles cases in 2025 being the highest since elimination, less than 1% of US counties had 20 or more cases [10].

Capitalising on the spatial heterogeneity of re-emergence risk, targeted preventive interventions and outreach may help mitigate the re-emergence of vaccine-preventable diseases if these locations can be accurately identified. Existing policy strategies to increase immunisation coverage are generally applied at larger spatial scales, such as state-level policy changes in the US that have banned nonmedical exemptions to childhood vaccine requirements and correspondingly increased vaccine coverage [11]. Similarly, while supplemental immunisation activities are a common and effective strategy to increase vaccination coverage in endemic settings [12], similar strategies may be considered in some re-emergent settings. However, because risk in re-emergence settings is spatially localised, interventions (e.g., vaccination campaigns, school outreach, involving community leaders) or particular policies can be targeted to the communities at greatest risk. Well-validated predictive models that can identify these areas of high risk will enable such pro-active localised interventions that can reduce outbreak and re-emergence risk.

Accurately assessing the locations at greatest risk of re-emergence to guide targeted interventions requires vaccine coverage and case data at a fine spatial scale. Because re-emergence risk is heterogeneous, national and even subnational vaccine coverage (e.g., state or provincial) obscure clusters of under-immunised individuals that can sustain transmission. Data at finer spatial and demographic resolutions (such as vaccine coverage by age group within a local municipality or school district level, and ideally stratified by sociodemographic characteristics) can enable characterisation of the sub-populations at greatest risk to inform and evaluate targeted outreach interventions. For instance, long-term tracking of spatially granular school-level vaccine coverage data in the US has revealed that, despite policy interventions, clusters of under-immunisation with particular geographic, socioeconomic, and demographic characteristics have persisted [13].

Nevertheless, the communities at greatest risk of re-emergence of vaccine-preventable disease are often those with significant vaccine hesitancy, underscoring the need to tailor intervention strategies to each individual and community. In any setting, the first step is to listen and understand concerns. At the patient-level, evidence-based strategies exist for clinicians to engage with patients about vaccines, including motivational interviewing, aligning on shared values (i.e., preventing illness of child), and discussing their concerns in detail (e.g., safety, vaccine approval process, ingredients, antigen load, etc.) [14]. On a population-level, strategies may include leveraging trusted leaders or public figures, engagement through social media platforms, and addressing documented barriers to vaccination. Efforts should focus on genuinely addressing concerns while simultaneously articulating the positive impact of vaccines to reduce illness and long-term complications, along with the safety of vaccines.

There remain unanswered questions around the re-emergence of vaccine-preventable diseases. We highlight three key areas of future work:

Firstly, what strategies will effectively address vaccine hesitancy to mitigate declining vaccination trends? While various approaches, ranging from patient-level to population-level interventions, have been successful in modestly increasing vaccine uptake, new approaches must be developed, evaluated, and applied. Such interventions should be tailored to be most effective in the particular communities and individuals as each context is unique.

Secondly, what are the unique epidemiologic patterns of re-emergence, and how can they be monitored to inform efforts to mitigate re-emergence risk? Key questions remain regarding how re-emergence risk evolves spatially over time and which populations are at greatest risk. Newer tools such as wastewater surveillance, genomic sequencing to distinguish importation from persistent transmission, and mobile app-based tools for contact tracing or reporting may facilitate these efforts, although their exact utility is case-dependent and evolving. Predictive models, when calibrated to detailed data and well-validated, may be valuable tools to integrate these diverse data streams and prioritise locations for targeted interventions.

Finally, what are scalable interventions and policy solutions to mitigate re-emergence risk at a population-level, and how do they vary by disease and setting? Although targeted interventions may be effective in the early stages of re-emergence, universal interventions may be necessary at later stages, and open questions remain around trade-offs between varying types of interventions. In some cases, addressing vaccine availability and access may be a key barrier. Furthermore, continued re-evaluation of vaccine recommendations across different re-emergence stages and by setting is important to optimise population-level protection. Re-emergence is a global challenge, and translating insights from endemic to re-emergent settings will be critical.

Whether the current re-emergence of vaccine-preventable diseases represents a temporary setback or the beginning of a broader transition remains to be seen, but even the existing trajectory represents a significant loss to decades of global health progress [5]. Nevertheless, re-emergence is currently in its early stages, and its heterogeneity presents an opportunity for targeted policy interventions that may effectively mitigate re-emergence. In an era where public health is increasingly politicised, continued investment and support for public health infrastructure is ultimately about preventing illness and saving lives. As the threat of re-emergence of vaccine-preventable diseases grows, much work remains to be done to sustain the value of vaccines worldwide.
